# Clinical features of neonatal hyperthyroidism: a retrospective analysis in southwestern China

**DOI:** 10.3389/fped.2024.1282408

**Published:** 2024-06-20

**Authors:** Yan Song, Hong Wei, Luying Cao, Pinglan Deng, Weixia Du, Shan Liu, Yu Zhang

**Affiliations:** ^1^ Department of Neonatology, Children's Hospital of Chongqing Medical University, National Clinical Research Center for Child Health and Disorders, Ministry of Education Key Laboratory of Child Development and Disorders, Chongqing, China; ^2^ Chongqing Key Laboratory of Pediatric Metabolism and Inflammatory Diseases, Chongqing, China; ^3^ Department of Nuclear Medicine, Children's Hospital of Chongqing Medical University, Chongqing, China

**Keywords:** neonatal hyperthyroidism, neonatal diseases, thyroid function, clinical analysis, clinical characteristics

## Abstract

**Purpose:**

This study aimed to explore the clinical characteristics and evaluate the different types of thyroid dysfunction in babies with neonatal hyperthyroidism.

**Methods:**

The clinical data of 19 neonates with hyperthyroidism admitted to the Children's Hospital of Chongqing Medical University between January 2012 and April 2021 were retrospectively analyzed.

**Results:**

Fifteen (78.9%) infants were born to mothers with Graves’ disease. Eleven (57.9%) infants were premature; two babies were born at small for gestational age. The age at diagnosis ranged from 3 to 34 days, with a mean of 18.53 ± 6.85 days. The majority of the babies presented with goiter (84.2%) and tachycardia (94.7%) after birth. Nine (47.4%) of them presented with abnormal weight gain, seven (36.8%) presented with stare or ocular protrusion, six (31.6%) presented with hyperexcitability, four (21.1%) presented with jaundice and liver dysfunction, two (10.5%) presented with sweating, one (5.3%) presented with fever, and one case presented without any symptoms. Transient hyperthyroidism was the main thyroid dysfunction in our study. Overt hyperthyroidism was diagnosed in 13 (68.4%) neonates. Another three babies (15.8%) presented with hyperthyroidism with slightly elevated free triiodothyronine levels, normal thyroxine (T4) levels, and low thyroid-stimulating hormone (TSH) levels. Normal thyroid hormone levels with low TSH levels were observed in three (15.8%) neonates. Ten children were treated with antithyroid drugs. Eighteen children recovered normal thyroid function at 1–3 months of age; one baby in the study group required further levothyroxine supplementation due to primary hypothyroidism (HT). One child was found to have developmental delay at 2 years of age during follow-up.

**Conclusions:**

Our study highlights the need for prolonged monitoring of thyroid function in suspected patients. A single normal screening for hyperthyroidism or the absence of a maternal history of hyperthyroidism cannot exclude this disease.

## 1 Introduction

Neonatal hyperthyroidism (NH) is a rare disorder in clinical practice, with a comprehensive incidence of about 1/50,000–2/50,000 (1). Most NH cases are transient; however, NH can lead to significant morbidity and mortality if not promptly recognized and adequately treated (2). Autoimmune thyroid disease (AITD) in mothers, during or before pregnancy, significantly increases the risk of NH, with an incidence of approximately 1%–5% (2–4). The diagnostic methods of NH have reached a clinical consensus. Early diagnosis and targeted treatment can effectively control the condition and reduce physical damage in children. However, neonatal hyperthyroidism is rare and can be easily misdiagnosed at an early stage; delayed diagnosis and treatment may affect the prognosis and quality of life of a newborn (5). At present, there are few reports on NH in China, and fewer than 100 cases have been reported in related studies. In this observational study, 19 cases of NH in the Children's Hospital of Chongqing Medical University were selected as the research subjects, and their clinical characteristics and the therapeutic effects of treatment were summarized to provide guidance for the early detection, diagnosis, disease evaluation, and treatment of NH. The thyroid dysfunction was evaluated to deepen the understanding of the clinical features of NH.

## 2 Patients and methods

### 2.1 Patients

Nineteen neonates with NH admitted to the Children's Hospital of Chongqing Medical University, the largest children's hospital in southwestern China, between January 2012 and April 2022 were retrospectively analyzed. All children were diagnosed with NH and received treatment and follow-up at the hospital. The diagnostic criteria are as follows (6): (1) medical history: whether the child's mother had a history of AITD; (2) clinical manifestations and signs: whether the child exhibited symptoms such as skin flushing, hyperphagia, diarrhea, slow weight gain (or no increase or even loss), irritability, sweating, tremor, periorbital edema, preference for eye-opening, lid lag, exophthalmos, increased heart rate, respiration or blood pressure, frontal bossing, goiter, hepatosplenomegaly, and microcephaly. These signs and symptoms were recorded when present, but they were not required for the final diagnosis.

Maternal data, including the history of the mother's thyroid disease, time of diagnosis (before or during pregnancy), and type of treatment [antithyroid drugs (ATDs) or other therapies], were collected.

The following neonatal data were analyzed: gestational age (GA), birth weight, Apgar score, and signs of hyperthyroidism. Infants were classified as small for gestational age (SGA) when their birth weights were below the 10th percentile for gestational age.

### 2.2 Treatment and follow-up

The times of normalization of thyroid-stimulating hormone (TSH), free thyroxine (fT4), and free triiodothyronine (fT3) levels and the treatment modality were analyzed in each child. ATDs were administered to the children as appropriate for their condition. Those with no obvious clinical signs and mild disease did not need to be treated, while those with obvious clinical signs or severe disease were treated with 0.25–1 mg/(kg day) of methimazole (MMI), or 5–10 mg/(kg day) of propylthiouracil (PTU). Children with hemodynamic instability received circulatory support, while those with respiratory distress were given respiratory support; adequate energy and fluid intake were ensured during treatment, and blood pressure, heart rate, liver function, routine blood tests, and thyroid function were regularly monitored. Children were discharged on medication once their symptoms were relieved or effectively controlled after hospitalization. It is recommended that the child return to the hospital every 1–2 weeks to re-examine the liver function and to evaluate the condition and efficacy of treatment. The type and dosage of drugs should be adjusted according to the patient's clinical manifestations and thyroid function indicators, and the dose of ATDs should be gradually reduced until complete drug withdrawal. During the follow-up period, the child's family members were instructed to continuously record the child's weight and other developmental parameters; additionally, they were instructed to assess the child's development when returning to the hospital for re-examination-. Each child continued to be followed up for 3 months after their thyroid function was completely normal.

## 3 Results

### 3.1 Maternal history of thyroid disease and perinatal conditions

The maternal and neonatal characteristics are presented in Table 1. In our study, 15 (78.9%) infants with NH were born to mothers with Graves’ disease (GD). Seven mothers were diagnosed before pregnancy [five of them were treated with ATDs, and two underwent radioactive iodine (RAI) therapy], and eight mothers were diagnosed with GD during pregnancy (seven in the first trimester and one in the third trimester). Three neonates, presented as cases 8, 10, and 12, were born to mothers diagnosed during pregnancy with hypothyroidism and treated with levothyroxine (L-T4). Only one neonate, presented as case 13, was born to a mother without a history of thyroid disease. Eleven (57.9%) of the infants were premature; two babies were born as SGA. In 52.3% of cases (10 out of 19), the delivery was by cesarean section.

**Table 1:** Maternal and neonatal characteristics.

Patient	Maternal thyroid disease history				Neonates				
	Time of diagnosis	Treatment before pregnancy	Treatment during pregnancy	Sex	GA (weeks)	Type of delivery	Apgar score	Weight (g)	Diagnostic day
1	GD (4 years)^a^	ATD	ATD	M	37 + 4	VD	9-10-10	3,350	18
2	GD (3 years)^a^	ATD	ATD	M	39 + 3	VD	10-10-10	3,450	24
3	GD (T1)		ATD	M	35 + 1	CS	9-9-10	1,850	34
4	GD (T1)		ATD	F	40 + 4	CS	10-10-10	3,590	14
5	GD (T3)		ATD	F	36 + 1	CS	10-10-10	2,350	18
6	GD (10 years)^a^	RI, L-T4	L-T4	F	34 + 1	VD	8-9-9	2,420	22
7	GD (5 years)^a^	RI, L-T4	L-T4	F	35 + 6	CS	8-9-10	2,590	27
8	HT (T1)		L-T4	M	32 + 6	VD	8-9-10	2,100	14
9	GD (T1)		ATD	F	37 + 4	VD	9-10-10	3,500	15
10	HT (T1)		L-T4	M	33 + 6	CS	8-9-9	2,200	22
11	GD (6 years)^a^	ATD, L-T4	L-T4	F	33 + 5	CS	8-9-9	2,080	26
12	HT (T1)		L-T4	M	36 + 2	VD	9-10-10	2,360	3
13	None			F	38 + 4	CS	10-10-10	3,400	14
14	GD (T1)		ATD	M	36	CS	9-10-10	2,740	13
15	GD (7 years)^a^	ATD	ATD	M	39	CS	10-10-10	3,700	22
16	GD (T1)		ATD	F	39 + 1	VD	10-10-10	2,550	17
17	GD (T1)		ATD	M	38 + 3	VD	10-10-10	2,620	14
18	GD (T1)		ATD	M	36 + 4	CS	9-10-10	2,500	22
19	GD (T1)		ATD	M	34 + 2	VD	9-9-9	2,100	13

yrs, years; T, trimester; RI, 131 iodine therapy; GW, gestational weeks; CS, cesarean section; VD, vaginal delivery; M, male; F, female.

^a^ Diagnosis before pregnancy.

### 3.2 Clinical manifestations

The age at diagnosis ranged from 3 to 34 days, with a mean of 18.53 ± 6.85 days. The children studied presented with varying degrees of NH. A majority of the babies presented with goiter (16/19, 84.2%) and tachycardia (18/19, 94.7%) after birth. Nine of them (47.4%) presented with abnormal weight gain, seven (36.8%) presented with stare or ocular protrusion, six (31.6%) presented with hyperexcitability, four (21.1%) presented with jaundice and liver dysfunction, two (10.5%) presented with sweating, one (5.3%) presented with fever, and one case presented with no symptoms.

### 3.3 Thyroid dysfunction treatment and outcomes

Table 2 shows the characteristics of thyroid function and the type of therapy in all the babies that were studied. Of the 19 babies, 13 (68.4%) (cases 1–6, 8–9, 14–15, and 17–19) showed overt hyperthyroidism; however, among them, six neonates with presented low TSH levels and normal thyroid hormone levels in the early days of life and did not require any pharmacological therapy. However, in the following few days, decreased TSH levels and high fT4 and fT3 levels were observed. Ten of these babies required ATD treatment, which was started between 3 and 47 days of life. The mean duration of ATD treatment was 37.2 days (range: 8–38 days). One baby in this group required further L-T4 supplementation (case 4, 2 months after the end of MMI treatment) and was diagnosed with primary hypothyroidism. Three of these babies received only propranolol therapy due to tachycardia for 23–57 days, with an initial dose of 2 mg/kg/day.

**Table 2:** Characteristics of thyroid function and the type of therapy in all the studied babies.

Patient	First thyroid blood test (start of treatment)				Thyroid blood test (end of MMI treatment)				Treatment duration (d)	Follow-up (growth and development)
	Age (d)	TSH (µIU/ml)	fT4 (pmol/L)	fT3 (pmol/L)	Age (d)	TSH (µIU/ml)	fT4 (pmol/L)	fT3 (pmol/L)		
1	18/37	0.012/<0.004	21.8/44.1	3.52/24.4	52	1.97	14.3	8.62	Propranolol (18–52)	Normal
2	24	<0.004	40.4	15.8	46	2.14	15.6	9.27	Propranolol (24–46) Propranolol (35–85)	Normal
3	34/47	0.051/0.005	32.6/>77.2	12.1/24.1	70	0.01	15.1	5.11	MMI (47–50) PTU (51–70)	Normal
4	14/32	0.098/0.006	49.9/45	14.3/26	43	1.97	14.3	8.54	Propranolol (14–43) MMI (32–43)	PH 2 months after MMI therapy
5	18	0.005	>77.2	25	45	0.017	14.3	8.25	Propranolol (21–77) MMI (21–45) Propranolol (22–44)	Normal
6	3/22	0.014/<0.004	13.5/>77.2	3.69/59	37	0.026	22.1	8	MMI (30–37) L-T4 (11–22)	Normal
7	27	<0.004	46	19.2	50	0.266	24.2	9.05	Propranolol (27–50)	Normal
8	8/14	0.008/<0.004	20.6/>77.2	8.05/36.2	21	<0.004	19	9.2	Propranolol (14–30) PTU (14–21)	Normal
9	26	0.015	>77.2	50.1	47	0.016	22	9.05	Propranolol (26–81) MMI (26–47)	Normal
10	8/22	0.014/<0.004	14.2/53.9	5.76/16.1	35	0.004	26.4	8.74	Propranolol (22–35)	Normal
11	26	0.008	41.6	14.9	40	0.028	25.2	10.2	Propranolol (26–40)	Normal
12	3	0.005	69	9.55	10	1.4	22.32	9.13	Observation	Normal
13	14	0.01	21.79	8.72	62	0.9	15.6	7.71	Propranolol (14–62)	Normal
14	13	0.009	>77.2	17.4	70	0.324	11.2	7.42	Propranolol (13–70) Propranolol (22–112)	Normal
15	7/22	0.02/0.014	26.8/40.4	16.1/15.8	60	0.008	11.7	5.78	MMI (22–60)	Mild delay
16	17	0.016	22.1	3.46	32	2.24	19.7	9.83	Propranolol (17–32)	Normal
17	14	<0.004	>77.2	36.2	28	0.006	25.2	9.65	Propranolol (14–43) MMI (14–28)	Normal
18	22	0.014	40.4	15.8	42	0.007	24.6	9.72	Propranolol (22–49) MMI (22–42)	Normal
19	13	0.009	>77.2	17.4	34	0.004	24.7	9.83	Propranolol (13–62) MMI (13–34)	Normal

d, day of life; PH, primary hypothyroidism.

Normal values [serum TSH (µIU/ml), fT4 (pmol/L), and fT3 (pmol/L) levels were measured by the chemiluminescence immunoassay method]: TSH: 0.33–8.6 µIU/ml; fT4: 1–7 days: 29.6–79.2 pmol/L, 8–30 days: 18–63.6 pmol/L, >30 days: 11.7–27.3 pmol/L; fT3: 1–7 days: 2.76–11.67 pmol/L, 8–30 days: 2.84–11.86 pmol/L, >30 days: 3.28–11.6 pmol/L.

Three children (15.8%, cases 7, 10, and 11) presented with hyperthyroidism with slightly elevated fT3 levels, normal thyroxine (T4) levels, and low TSH levels. All three babies received only propranolol therapy due to tachycardia. One of them (case 10) was born to a mother diagnosed with hypothyroidism during pregnancy. The other two babies were born to mothers who had a history of GD before pregnancy and developed hypothyroidism after definitive therapy with RAI.

Normal thyroid hormone levels with low TSH levels were observed in three neonates (15.8%, cases 12, 13, and 16). One of them, born to a mother diagnosed with hypothyroidism during pregnancy (case 13), did not require pharmacological therapy. Two babies received propranolol therapy due to transient tachycardia; one neonate was born to a mother without thyroid disease. In one of them (case 13), low TSH levels continued through the first 2 months of life and then spontaneously normalized.

All 19 children were discharged after 6–26 days of hospitalization, and 18 of them recovered normal thyroid function at 1–3 months of age. One baby required further L-T4 supplementation 2 months after the end of MMI treatment and was diagnosed with primary hypothyroidism. One full-term child was found to have developmental delay at 2 years of age during follow-up.

## 4 Discussion

Neonatal hyperthyroidism (NH) is mainly related to the transplacental passage of maternal antithyrotropin receptor antibodies (TRAbs) (7–9). During pregnancy, TRAbs freely cross the placenta and either overstimulate the thyroid-stimulating antibody (TSAb) or block the thyroid blocking antibody (TBAb) in the fetal thyroid gland (10, 11). This study retrospectively analyzed the clinical data of 19 infants diagnosed with hyperthyroidism in the neonatal period. As expected, the majority (78.9%) of babies were born to mothers with GD, as reported in other studies (12), although two of the mothers with a history of GD developed hypothyroidism after RAI therapy and were treated with L-T4 during pregnancy. Therefore, infants born to mothers with GD, even those mothers with hypothyroidism following definitive therapy with RAI or a total thyroidectomy, should undergo thyroid function screening because increased TRAb levels can persist for years after definitive therapy (13, 14). However, in our study, three neonates were born to mothers diagnosed during pregnancy with hypothyroidism. Similar cases were described in the literature (15, 16).

Maternal GD may cause premature birth and intrauterine growth restriction (17), particularly in cases where maternal GD is poorly controlled. In our study, 11 (57.9%) infants were premature and 2 babies were born as SGA. The clinical manifestations of NH are diverse and lack specificity. The babies affected may present with tachycardia, hyperexcitability, poor weight gain, fever, and other hypermetabolic manifestations, as well as systemic manifestations involving the cardiovascular, respiratory, digestive, blood, and other systems (18, 19). These unspecific signs of hyperthyroidism may be confused with infection/sepsis; therefore, the diagnosis of NH is often delayed (20), especially when the mother has no apparent history of thyroid disease. The mean age at diagnosis in our cases is 18.53 ± 6.85 days. The later diagnosis and initiation of treatment may have contributed to the more typical clinical presentation of hyperthyroidism in our cases.

NH is not the only consequence of maternal GD. Thyroid function disturbances observed in newborns depend on the type and level of maternal antibodies. The administration of ATDs during pregnancy could also impact neonatal thyroid function (10). Due to different levels and clearance rates of maternal ATDs and TRAb, the typical biochemical changes of hyperthyroidism may be delayed. We observed that 6 of the 13 babies with overt hyperthyroidism presented with low TSH levels and normal thyroid hormone levels in the first days of life. However, in the following few days (14–47 days after birth), decreased TSH levels and high fT4 and fT3 levels were found. Neonates with maternal GD may also develop transient hypothyroidism in the first days of life (21, 22). One case in our study (case 10) was diagnosed with hypothyroidism on the eighth day of life and was administered L-T4, but hyperthyroidism was observed on the 13th day. Therefore, even if the initial thyroid function tests are normal or suggest hypothyroidism, there is a need for the prolonged monitoring of thyroid function in the offspring of mothers with GD.

Appropriate treatment should be started immediately after the diagnosis of NH. With the clearance of maternal TRAb from the circulation, symptoms of NH will generally resolve in 4–6 months after birth (23). In our study, 18 (94.7%) patients recovered normal thyroid function at 1–3 months of age, while one baby required further L-T4 supplementation due to primary hypothyroidism. During ATD treatment, thyroid function tests should be monitored closely every 1–2 weeks.

The prognosis of NH depends on the duration and severity of hyperthyroidism. Studies on neurocognitive outcomes of newborns with hyperthyroidism are limited. Daneman and Howard reported on eight children with histories of NH, four of whom showed intellectual impairment at the age of 2 years or older (24). Another study showed no significant difference in physical and intellectual development between neonates with hyperthyroidism and normal children when they were followed up to 7–8 years old (25). We observed a one full-term child who had a developmental delay at 2 years of age. However, due to the fact that some children in our retrospective study were too young at the time of follow-up to undergo a full evaluation of their developmental outcomes, including a comprehensive psychometric evaluation, there remains the potential for further developmental challenges to arise as they grow older. Therefore, long-term follow-up of newborns with hyperthyroidism is needed.

The main limitation of the present analysis is the small size of the study group. In addition, all cases were transferred from other hospitals, resulting in incomplete collection of data on maternal and fetal conditions (especially maternal TRAb concentration).

## 5 Conclusion

In summary, NH is rare in clinical practice and presents with atypical early symptoms, posing a high risk of misdiagnosis in the early stages of the disease. Continuous monitoring of thyroid function in suspected patients is the key to managing neonatal hyperthyroidism. A single normal screening or the absence of a maternal history of hyperthyroidism cannot exclude this disease.

## Data Availability

The original contributions presented in the study are included in the article/Supplementary Material, further inquiries can be directed to the corresponding author.
